# Seedling Establishment of Tall Fescue Exposed to Long-Term Starvation Stress

**DOI:** 10.1371/journal.pone.0166131

**Published:** 2016-11-10

**Authors:** Antonio Pompeiano, Claudia Roberta Damiani, Sara Stefanini, Paolo Vernieri, Thais Huarancca Reyes, Marco Volterrani, Lorenzo Guglielminetti

**Affiliations:** 1 Laboratory of Ecological Plant Physiology, Global Change Research Institute CAS, Brno, Czech Republic; 2 Federal University of Grande Dourados, Dourados, Brazil; 3 Department of Agriculture, Food and Environment, University of Pisa, Pisa, Italy; Chinese Academy of Sciences, CHINA

## Abstract

In germinating seeds under unfavorable environmental conditions, the mobilization of stores in the cotyledons is delayed, which may result in a different modulation of carbohydrates balance and a decrease in seedling vigor. Tall fescue (*Festuca arundinacea* Schreb.) caryopses grown at 4°C in the dark for an extended period in complete absence of nutrients, showed an unexpected ability to survive. Seedlings grown at 4°C for 210 days were morphologically identical to seedlings grown at 23°C for 21 days. After 400 days, seedlings grown at 4°C were able to differentiate plastids to chloroplast in just few days once transferred to the light and 23°C. Tall fescue exposed to prolonged period at 4°C showed marked anatomical changes: cell wall thickening, undifferentiated plastids, more root hairs and less xylem lignification. Physiological modifications were also observed, in particular related to sugar content, GA and ABA levels and amylolytic enzymes pattern. The phytohormones profiles exhibited at 4 and 23°C were comparable when normalized to the respective physiological states. Both the onset and the completion of germination were linked to GA and ABA levels, as well as to the ratio between these two hormones. All plants showed a sharp decline in carbohydrate content, with a consequent onset of gradual sugar starvation. This explained the slowed then full arrest in growth under both treatment regimes. The analysis of amylolytic activity showed that Ca^2+^ played a central role in the stabilization of several isoforms. Overall, convergence of starvation and hormone signals meet in crosstalk to regulate germination, growth and development in tall fescue.

## Introduction

In higher plants, sugars function as metabolic energy storage and structural cellular components, but they also serve as regulators of plant growth and development. Although plants are largely considered carbon autotrophs, they can be viewed as carbon heterotrophs during certain stages of their life cycle, e.g. senescence, postharvest period or seed germination [1]. Energetically inefficient breakdown of carbohydrates may quickly drain the carbohydrate stores in sink tissues during night [2], as well as when plants may be exposed to biotic or abiotic stresses that affect photosynthetic efficiency in source tissues [3]. A lack of sugar can induce substantial physiological and biochemical changes aimed at sustaining respiration and metabolic processes, due to a complex sugar-sensing network. Sugar starvation generally triggers the following sequential cellular events: (a) arrest in cell growth, (b) rapid consumption of cellular carbohydrate and decrease in respiration rate, (c) degradation of lipids and proteins, (d) increase in accumulation of phosphate, phosphorylcholine and free amino acids, and (e) decline in glycolytic enzymatic activities [1].

Sugars also interact with phytohormones to regulate seed germination via intricate pathways. Among the phytohormones that are key regulators of germination, gibberellin (GA) and abscisic acid (ABA) have the most pronounced effects. The dormant state is characterized by increased ABA biosynthesis and GA catabolism, and thus, seed dormancy release and germination are especially related to the ratio of GA to ABA. [4, 5].

Important environmental cues that alter seed dormancy and germination include water, oxygen and temperature. Seeds may also be sensitive to additional factors such as light quality and quantity, and nutrient availability [6]. Temperature plays an important role in controlling seed germination rates and timing. Based on patterns of change in physiological responses to temperature, five types of non-deep physiological dormancy (PD) can be identified. Most seeds belong to type 1, in which the range of temperatures at which germination can occur increases gradually during the progression of non-deep dormancy release from low to high (e.g. *Arabidopsis thaliana*). Moreover, temperature, a factor related to slow seasonal change, is integrated over time to alter the depth of dormancy and the sensitivity of other factors (e.g. light) [7].

Germination and subsequent seedling growth rely on an efficient primary metabolism for the mobilization of seed reserves. Starch, which makes up ~75% of the endosperm by dry weight in cereal grains (proteins, lipids, nucleic acids, and mineral complexes), is degraded and mobilized by a series of enzymes such as α-amylase (α-1,4 glucan-4-glucanohydrolase; EC 3.2.1.1). Sugar signal transduction and gene regulation are central control mechanisms that mediate energy homeostasis, carbohydrate distribution, and the growth and development of plants. A good example of this phenomenon is the regulation of the α-amylase genes in germinating cereal caryopses, where α-amylase genes are induced by GA and by increased sugar demand/sugar starvation, while are repressed by ABA. In barley, α-amylase synthesis is also regulated by calcium, which is required to maintain amylases activities and the thermostability in the endoplasmic reticulum of the aleurone layer where these enzymes are synthesized. Moreover, the irreversible binding of calcium to amylase stabilizes its secondary and tertiary structures without influencing its primary structure [8].

Tall fescue (*Festuca arundinacea* Schreb.), a cool-season bunch-type grass, is a wise choice for stockpiling because it maintains its nutritional value longer into winter than many other forage crops [9]. Its native range encompasses a geographical area that stretches from the Mediterranean coasts to the high altitudes of the Alps and the northern latitudes of continental Europe. This wide distribution is possible thanks to its ability to survive in a wide range of soils, climates, and management conditions [10]. In response to temperature changes, the allohexaploid genome of this species changes the numbers of interspersed DNA repeats when seedlings are exposed to 10°C as opposed to 30°C [11]. Such genome plasticity allows this species to achieve optimal growth dynamics at different developmental stages and in different environments, which may explain, at least in part, the ability of this species to survive over such a wide geographical area [11, 12].

The major factors limiting tall fescue are climatic and geographic, rather than edaphic or biotic [13]. A number of studies have been conducted on how tall fescue germination, growth and development is affected by limiting temperatures [14–20]. Knowledge of the response to sugar starvation and adaptation mechanisms in plants is a basic research interest as well as having important agronomic implications. In this study, we assess the influence of temperature (23°C or 4°C) on tall fescue seedling establishment under nutritional stress. To achieve this objective, we carried out a time-course experiment to verify the ability of the caryopses to complete germination under prolonged cold temperatures in complete absence of light and nutrients. We also analyzed the temperature-driven changes at physiological and molecular level. Examination of photosynthetic tissues in plants grown for 400 d under cold conditions hinted at the ability to rapidly restore photosynthetic activity upon illumination. We then examined whether an alternative stress acclimation process exists over the long term that involves a decrease in storage compound hydrolysis, a drastic reduction of growth and amylases stabilization by mobilization of calcium molecules.

## Materials and Methods

### Plant material and growth conditions

Caryopses of *Festuca arundinacea* Schreb., cv. Arminda, (Barenbrug Holland B.V.) were sterilized in sodium hypochlorite solution (5% active chlorine) for 10 min. After surface sterilization, caryopses were rinsed ten times in sterile water, placed in Petri dishes (9 cm diameter) containing 10 mL sterile bi-distilled water and dishes were sealed with parafilm. Growth behaviour and physiological parameters were recorded in growth chamber under two different temperature regimes, 23°C or 4°C, both in the dark. Temperature monitoring was carried out with a data logger (Campbell Scientific, Logan, UT, USA), and relative humidity was monitored with a hygrothermograph in each chamber. Three biological repeats were performed for each experimental condition, and all treatments were performed in parallel. For each combination of treatment and target time (until 21 and 400 d, at 23 and 4°C, respectively, as reported in figures), samples were collected and immediately processed or ground in liquid nitrogen and stored at –80°C for further molecular and biochemical analyses.

### Chemicals

Kits for protein determination were purchased from Bio-Rad Laboratories (Hercules, Ca, USA), kits and gels for electrophoresis were purchased from Amersham Biosciences (Uppsala, Sweden), and all the other reagents used were purchased from Sigma (St. Louis, Mo, USA).

### Histological analysis

The anatomy of tall fescue seedlings grown for 400 d at 4°C was analyzed and compared with that of seedlings grown for 21 d at 23°C. Subsequently, the seedlings were transferred from the dark to light for 3 d of recovery (23°C, 12 h photoperiod at 150 μmol m^–2^ s^–1^ photosynthetic photon flux density (PPFD) provided by a combination of incandescent and fluorescent lamps) and resulting anatomical changes were recorded. The anatomical sections were prepared by hand using fresh material. Segments were stained with Giemsa (methylene blue) solution, and 80% glycerol (commercial solution) was added for semi-permanent preservation of the histological sections. After staining, segments were analysed by optical microscopy.

### Abscisic acid quantification

ABA (free form) was determined in crude aqueous extracts of plant material by solid phase radioimmunoassay (RIA) using the monoclonal antibody DBPA-1 [21, 22], which proved to be highly specific for S(+)-ABA [23]. Plant samples were weighed, ground in liquid nitrogen and extracted with 0.5 mL of distilled water overnight at +5°C in the dark. Next, 50 μL aliquots were assayed in triplicate by RIA. Validation of RIA results on tall fescue seedling tissues by HPLC fractionation of crude extracts and by internal standardization experiments was as previously described [24].

### Gibberellic acid bioassay

Samples were homogenized in ice-cold 80% (v/v) aqueous methanol and stirred for 12 h at 4°C. Samples were centrifuged at 14,000x g for 15 min. The pellets were separated from the supernatant and re-extracted twice. The combined supernatants were reduced to the aqueous phase and the pH adjusted to 3.0. The aqueous phase was then partitioned four times against water-saturated ethyl acetate (EtOAc). The EtOAc extracts were dried by evaporation and the samples were resuspended in water. Gibberellins were quantified by dwarf-rice bioassay (*Oryza sativa* L., cv. Tan-Ginbozu) as previously described by Jones and Varner [25].

### Starch analysis

Endosperm samples (100 mg fresh weight, FW) were ground in a mortar, resuspended in 100 mL of 10 mM KOH and boiled for 1 min. 1 mL of 1 N HCl was then added to each sample. The starch standard solution was prepared using 100 mg of potato soluble starch dissolved in 100 mL distilled water and boiled 1 min. Aliquots of samples (50 μL) and standards (from 0 to 100 μL) were adjusted to 150 μL with distilled water, and 1 mL of fresh iodine solution (0.13% K_2_ and 0.3% KI, dissolved in distilled water) was added. The absorbance was immediately read at 595 nm. Starch concentration was expressed as mg starch g^–1^ FW.

### Nonstructural carbohydrates quantification

Endosperm, coleoptile and root samples (100 mg FW) were ground to a powder in liquid nitrogen and extracted as described by Tobias et al. [26]. Samples were assayed using coupled enzymatic assay methods [27] that measure the increase in A_340_. Recovery experiments were carried out to evaluate loss during extraction. Two tests were performed for each metabolite by adding a known amount of authentic standards to the samples before proceeding with the extraction. The concentrations of the standards added were similar to those estimated to be present in the tissues in preliminary experiments. The percentage of recovery ranged between 96 and 104% depending on the sugar. The quantity of soluble carbohydrates was corrected on the basis of the recovery percentages for each sample, and expressed as μmoles hexose equivalents g^–1^ FW.

### Protein extraction for enzyme activity

Samples obtained from *F*. *arundinacea* endosperm treated as previously described (see Plant material and growth conditions) were extracted in 5 vol. of 100 mM HEPES-KOH pH 7.5, containing 1 mM EDTA, 5 mM MgCl_2_, 5 mM DTT, and 10 mM NaHSO_3_. Extracts were centrifuged (13,000x *g*, 15 min), the resulting pellets were washed with the extraction buffer, centrifuged again and the resulting supernatants were used for the enzymatic assays.

### Enzyme activity

Samples were assayed at 25°C in 0.5 mL reaction mixtures, following the methods previously described by Guglielminetti et al. [28]. Amylolytic activity was assayed using tall fescue crude extracts and crude extracts heated at 70°C for 15 min in the absence or presence of 3 mM CaCl_2_. To indirectly test whether the stabilization factors found in long-term starved tall fescue amylases could also stabilize the amylolytic activity in other plant species, we used barley endosperm extract together with tall fescue extract taking in advantage the non-overlapping isoelectric points for amylase isoforms. Enzyme activity was also investigated on tall fescue samples mixed with barley endosperm extracts obtained from caryopses germinated for 7 d in sterile bi-distilled water at 23°C in the dark. When extracts were composed from tall fescue and barley, amylolytic activity was assayed using also 2 min boiled-treated or dialyzed tall fescue extracts according to Guglielminetti et al. [29].

### Zymogram of total amylolytic activity

Isoelectric focusing (IEF, pH range 4.5–5.5) and activity staining of equal amount (10 μg) of proteins were performed on tall fescue extracts according to Perata et al. [30, 31]. When tall fescue and barley extracts were mixed, pH range 6.25–7.25 was used and the amount of protein used in a single lane was 6 μg (2 μg tall fescue mixed with 4 μg barley).

### Statistical analysis

Statistical analysis of biometric and physiologic traits was performed using two-way analysis of variance (ANOVA). When significant differences were found, the means were compared using the least significant difference (LSD) test. Significant differences for all statistical tests were evaluated at the level of *p* = 0.05. All computations were performed with R 3.2.3 [32], and the R package *agricolae* [33] was used.

## Results

For all experiments, morphological analogous seedlings were paired based on biometric traits in order to compare the effects of the treatment. This was necessary because the growth of seedlings incubated at 4°C was approximately ten times slower than those grown at 23°C.

### Shoot and root elongation

**Fig 1:**
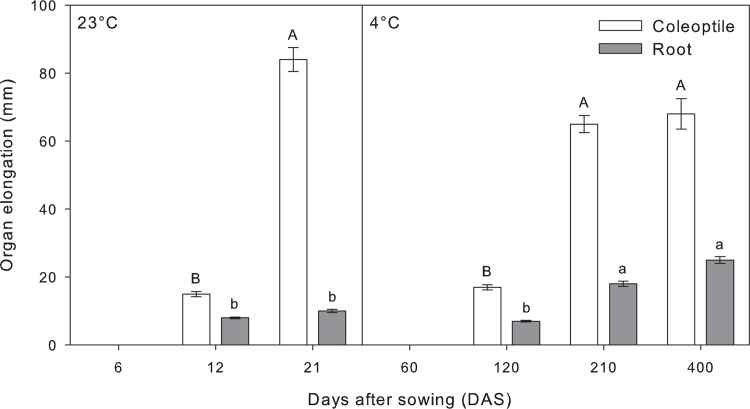
Tall fescue (*Festuca arundinacea* Schreb.) coleoptile and root elongations recorded in the time-course experiment at 23°C and 4°C. Within organ, bars followed by the same letter are not statistically different according to Fisher’s protected LSD (α = 0.05). Data are the mean of three experiments ± SE.

Changes over time in coleoptile and root length in response to both temperature conditions were recorded (Fig 1, S1 Table). Elongation of both organs began 12 days after sowing (DAS) at 23°C (control conditions), while it was delayed until 120 DAS in seedlings grown at 4°C. Both organs elongation of seedlings grown at 4°C had significantly increase at 210 DAS in comparison with that of 120 DAS. Seedlings grown under control conditions showed differences in the elongation of organs at 21 DAS, while coleoptile showed a high increase in comparison to 12 DAS, root length was maintained. Further slight, but not significant, coleoptile and root elongations were recorded at 400 DAS when seedlings grown at 4°C.

### Histological analysis

**Fig 2:**
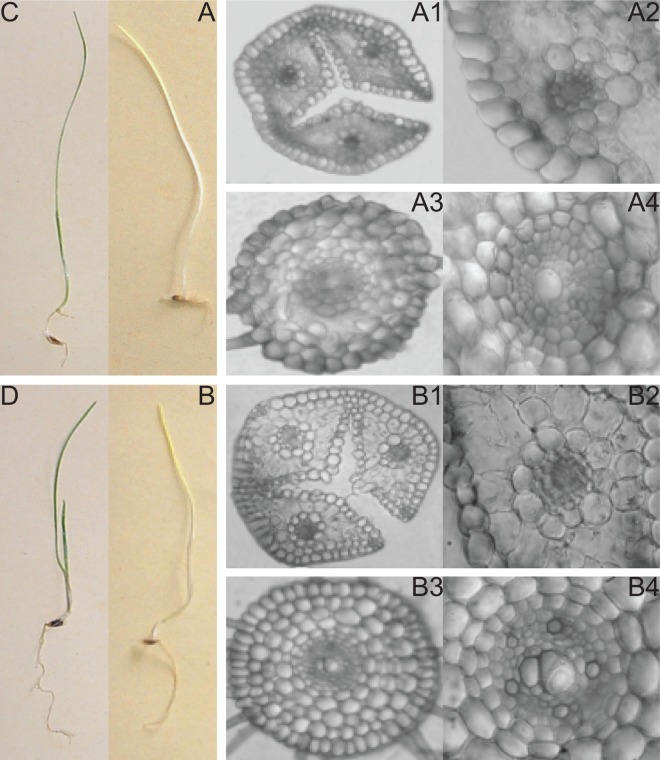
A and B. Representative seedlings of tall fescue (*Festuca arundinacea* Schreb.) grown under dark condition for 21 DAS at 23°C (A) and 400 DAS at 4°C (B). C and D. Seedlings response to the light after 3 d of recovery (23°C, 12/12 h photoperiod at 150 μmol photons m^–2^ s^–1^) in tall fescue from control (C) and cold (D) conditions. A1 and A2. Longitudinal section of tall fescue leaf grown under dark condition for 21 DAS at 23°C. A3 and A4. Longitudinal section of tall fescue root grown under dark condition for 21 DAS at 23°C. B1 and B2. Longitudinal section of tall fescue leaf grown under dark condition for 400 DAS at 4°C. B3 and B4. Longitudinal section of tall fescue root grown under dark condition for 400 DAS at 4°C.

The anatomy and morphology of tall fescue seedlings grown in complete darkness were carefully compared at the last time point: 21 DAS at 23°C *vs*. 400 DAS at 4°C (Fig 2A and 2B). The overall morphology and internal cellular organization of both coleoptile and root tissues in plants grown under control conditions for 21 d were strikingly similar to that of tissues found in plants grown at 4°C for 400 d, although cold-growth seedlings had a better organized cellular structure, with the presence of a regular concentric arrangement of cell files (Fig 2A and 2B).

Under control conditions, plastids differentiated into etioplasts and showed pronounced vascular development. Microscopic results in coleoptile tissue included xylem fiber differentiation and expansion, and thin cell wall delimiting the epidermis (Fig 2, A1-2). In root tissue, unthickened endodermis was observed (Fig 2, A3-4). Under cold growth condition, atypical anatomical adaptations were observed in etiolated plants. Microscope results in coleoptile tissue included an increase in cell wall thickness, the presence of undifferentiating plastids and some less developed xylem vessels (Fig 2, B1-2). In root tissue, an increased number of root hairs was observed (Fig 2, B3-4).

At the end of the treatment, plants from each experimental condition were re-exposed to the light in order to evaluate their recovery (Fig 2C and 2D). Tall fescue plants grown under cold condition displayed normal anatomy after recovery showing similar phenotype to plants grown under standard conditions. Moreover, cold stressed plants upon illumination showed re-differentiation of plastids into photosynthetically active chloroplasts, development of bulliform cells in the leaf epidermal layer, and a decline of root hairs (data not shown).

### GA and ABA endogenous levels

**Fig 3:**
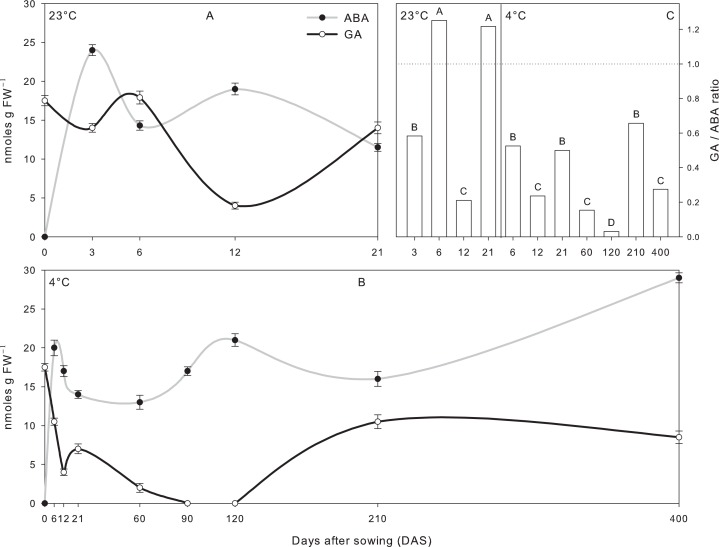
A and B. Endogenous level of ABA and GA in tall fescue (*Festuca arundinacea* Schreb.) caryopses grown under dark condition recorded during the time-course experiment at 23°C (A) and 4°C (B). C. Ratio of endogenous levels of GA and ABA in tall fescue caryopses grown under dark condition recorded during the time-course experiment at 23°C and 4°C. Bars followed by the same letter are not statistically different according to Fisher’s protected LSD (α = 0.05). Data are the mean of three experiments ± SE.

**Fig 4:**
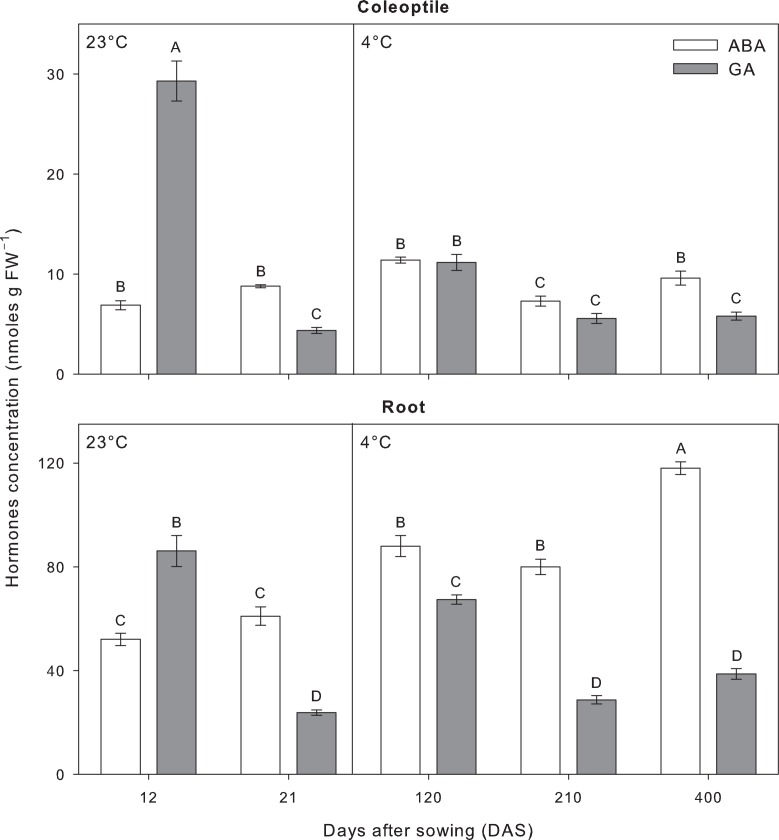
Endogenous level of ABA and GA in coleoptile and root tissues of tall fescue (*Festuca arundinacea* Schreb.) grown under dark condition recorded during the time-course experiment at 23°C and 4°C. Bars followed by the same letter are not statistically different according to Fisher’s protected LSD (α = 0.05). Data are the mean of three experiments ± SE.

To investigate the role of hormones in controlling growth under the different temperature regimes, endogenous levels of GA and ABA were analyzed in caryopses, coleoptile and root tissues during the time-course experiment (Figs 3 and 4).

In caryopses tissues, GA and ABA hormones showed a specular trend in each temperature treatments (Fig 3A and 3B). Despite the different time scale, the phytohormones profiles proved comparable when normalized to physiological status, where both the onset and the completion of germination could be linked to the GA/ABA ratio. Thus, under control conditions, germination was induced at 12 DAS when ABA levels were much lower respect to GA (Fig 3A) resulting in the minimum value of GA/ABA ratio (Fig 3C). Similarly, germination under cold temperature occurred simultaneously with lowest GA/ABA ratio recorded at 120 DAS (Fig 3C). Moreover, the hormonal pattern at 4°C showed that as exposure time was prolonged, an increase in GA levels was associated with progression towards germination completion (Fig 3B). Hormonal analysis in caryopses tissues showed evident differences between temperature regimes, two time points with GA/ABA ratio >1 were registered at 23°C, while all GA/ABA ratios at 4°C did not rise to 1 (Fig 3C).

GA and ABA levels analyzed in coleoptile and root tissues are shown in Fig 4. In seedlings grown at 23°C, phytohormone levels showed a similar pattern in both tissues with a sharp decline of GA at the last data point (–85.1% in coleoptile and –72.3% in root tissues) (Fig 4). A paired GA and ABA levels was found in coleoptiles grown at 4°C at 120 and 210 DAS, although a significant decline of GA respect to ABA was recorded at the last data point (Fig 4). In roots of plants grown at 4°C, a non-paired GA and ABA levels was observed with significant lower concentrations of GA throughout the experimental period (Fig 4).

### Starch content

**Fig 5:**
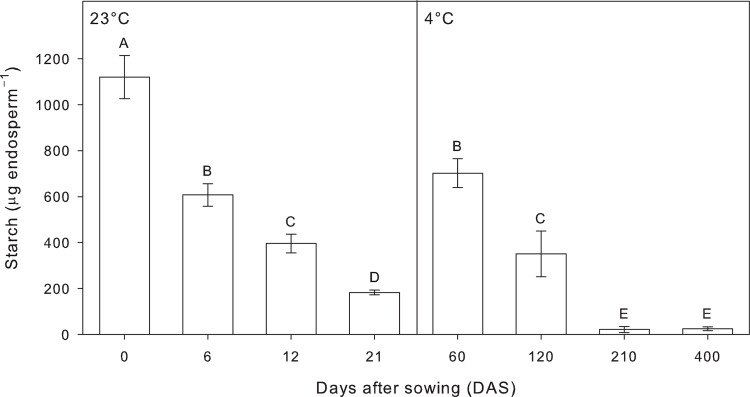
Starch content in tall fescue (*Festuca arundinacea* Schreb.) caryopses recorded in the time-course experiment grown under dark condition at 23°C and 4°C. Bars followed by the same letter are not statistically different according to Fisher’s protected LSD (α = 0.05). Data are the mean of three experiments ± SE.

Starch reserves significantly declined upon the onset of the treatment, with a continuously decrease during the time-course experiment under both temperature regimes (Fig 5). Starch content in plants grown at 23°C for 6 d showed no significant differences to that of plants grown at 4°C for 60 d, in both cases represented a decline of approximately 41.6% starch respect to the time 0. Similar situation was observed in the second time point (12 and 120 DAS) under both temperatures regimes, with a decline of approximately 66.7% starch respect to the time 0. Plants grown at 23°C for 21 d showed a decrease of approximately 83.7% starch respect to the time 0, while the starch content in plants at 4°C for 210 d showed almost a complete depletion. This low starch content in plants grown at 4°C was maintained up to the last time point, 400 DAS, which represented a decreased of approximately 98% respect to the time 0.

### Nonstructural carbohydrates

**Fig 6:**
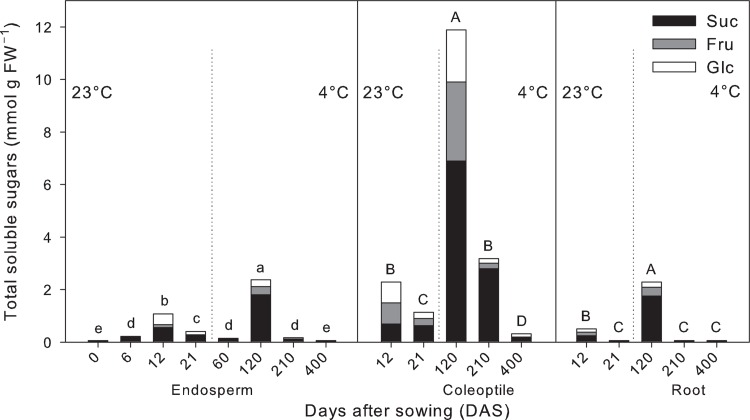
Total soluble sugars content in endosperm, coleoptile, and root tissues of tall fescue (*Festuca arundinacea* Schreb.) seedlings. Glucose (Glc), fructose (Fru), and sucrose (Suc) were assayed during the time-course experiment at 23°C and 4°C. For each organ, bars followed by the same letter are not statistically different according to Fisher’s protected LSD (α = 0.05). Data are the mean of three experiments.

Total soluble sugars (TSS) content varied with the organs of tall fescue plants. However, independently of the treatment, higher concentrations of soluble carbohydrates were found in the coleoptile, reaching a maximum of approximately 12 mmol g^–1^ FW at 120 DAS under cold conditions (Fig 6). Carbohydrate levels in the endosperm, coleoptile, and root also varied with the treatment, where the differences became more pronounced when exposure time increased (Fig 6). In all three organs, greater carbohydrate abundance was observed in cold treated plants, but with a faster depletion of reserves in comparison with that of plants grown under control conditions (Fig 6). Under control conditions, the maximum sugar reserves in all organs were obtained at 12 DAS, following by a considerable decline at 21 DAS when the roots exhibited greater carbohydrate shortage (– 88%) than the endosperm (-62%) and coleoptile (–50%). In response to low temperature, the maximum TSS in all organs were obtained at 120 DAS, following by a decline at 210 DAS when the endosperm (–93%) and roots (–98%) showed a sharp and more pronounced decline than coleoptile (–73%). Prolonged low temperature stress (400 DAS) showed the lowest TSS level with 0.319 mmol g^–1^ FW in the coleoptile and approximately 0.058 mmol g^–1^ FW in endosperm and roots (Fig 6).

Among the individual soluble carbohydrates that contributed to the above-described changes in TSS content between treatments, sucrose (Suc) was the most abundant representing 62% of the TSS on average, while content of glucose (Glu) and fructose (Fru) typically occurred in an approximately 1:1 ratio (Fig 6). During seedling emergence, Fru was the most responsive component in all tissues under both temperature regimes with a depletion averaging 83% at 23°C and 96% at 4°C. On the contrary, Suc was the least responsive sugar with a depletion averaging 50% at 23°C and 16% at 4°C (Fig 6).

### Total amylolytic activity

**Fig 7:**
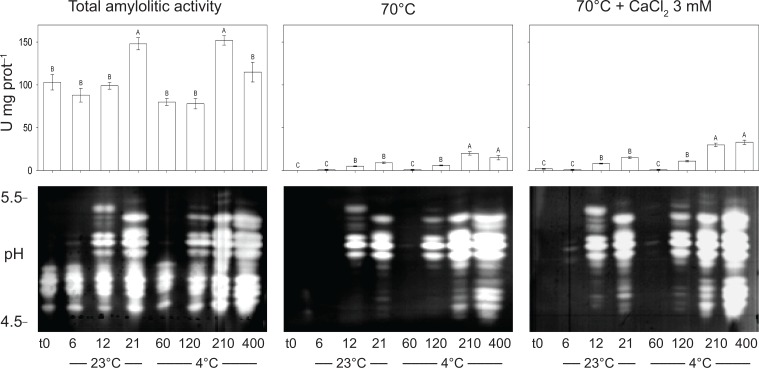
Amylolytic activities in tall fescue (*Festuca arundinacea* Schreb.) caryopses grown under dark at 23°C and 4°C. Data are the mean of three repetitions ± SE. Bars followed by the same letter are not statistically different according to Fisher’s protected LSD (α = 0.05). A representative isoforms pattern of amylase activities is shown below each respective bar plot. Crude extracts or heat-treated (70°C for 15 min in the absence or presence of 3 mM CaCl_2_) were loaded in equal amount on IEF and detected by activity staining.

Amylolytic activity was analyzed in tall fescue caryopses grown at 23 and 4°C during a time-course experiment. Results showed that several isoforms, which were absent in the control samples, were identified in cold treated seedlings (Fig 7). The total amylolytic activity was not significantly affected at the beginning of the time-course experiment in both temperature conditions when compared to the time 0 (Fig 7, left panels). Under control conditions, total amylolytic activity reached its maximum at 21 DAS with an increase of 43.7% respect to the time 0. Similar pattern was observed under cold conditions where the total amylolytic activity reached its maximum at 210 DAS, following by a decrease at 400 DAS (–24.3%, *P* < 0.001). No differences in total amylolityc activity were detected when samples paired by analogous morphological status were compared (6, 12 and 21 DAS *vs*. 60, 120 and 210 DAS at 23 and 4°C, respectively) (Fig 7, left panels).

In order to determine the presence of stabilized α-amylase by endogenous Ca^2+^, samples were assayed using heat-treated crude extracts. Almost no activity was detected at 6 DAS at 23°C and 60 DAS at 4°C, similar to the time 0 (Fig 7, middle panels). However, the levels were increased in the following time points under both temperatures regimes, with highest increase at 210 or 400 DAS under cold stress (Fig 7, middle panels). Further analysis was performed to determine the contribution of α-amylase in total amylolytic activity, where crude extracts were treated at 70°C in the presence of CaCl_2_ to inactivate ß-amylase, debranching enzyme and α-glucosidase (Fig 7, right panels). It was observed that α-amylase activity was low at 6 DAS at 23°C and 60 DAS at 4°C, following by an increase in both temperature regimes. Increased α-amylase activity reported in seedlings at 12 DAS under control conditions showed no difference with that of seedlings at 21 DAS. Under cold stress conditions, high α-amylase activity was reported at 120 DAS, which was comparable to that of 12 DAS at 23°C, following by a significant increase at 210 DAS. Slight but not significant increase in α-amylase activity was detected at 400 DAS in comparison to 210 DAS under cold stress (Fig 7, right panels). In addition, we observed that α-amylase activity negatively correlates with starch content (*r*<–0.847, *p*<0.01, data not shown).

### Tall fescue α-amylase stabilizing factors

Since we previously found that stable amylase isoforms were induced by prolonged exposure to cold stress (210 and 400 DAS) (Fig 7), we examined whether the stabilization factors found in the tall fescue extracts would be able to stabilize the amylolytic activity in other plant species. Barley (*Hordeum vulgare* L.) was selected to perform this experiment taking in advantage its non-overlapping isoelectric points for amylase isoforms (pH 6.25–7.75) in amylolytic activity respect to that of tall fescue (4.50–5.50).

**Fig 8:**
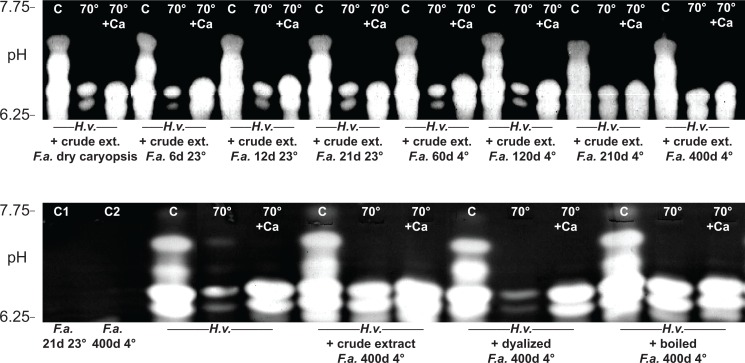
Zymograms of amylolytic activities from barley extracts blended with tall fescue (*Festuca arundinacea* Schreb.) extracts. C corresponds to total amylolytic activity of barley (*H*.*v*.) crude extracts. C1 and C2 correspond to tall fescue (*F*.*a*.) crude extracts at 21DAS (23°C) and 400 DAS (4°C), respectively. Crude extracts and heat-treated 70°C for 15 min (70°) or 70°C for 15 min with 3 mM CaCl_2_ (70°+Ca) were loaded in equal amount on IEF and detected by activity staining.

Extracts from tall fescue seedlings grown at 4°C for at least 210 d substantially stabilized barley amylases, and a pronounced effect was also detected using extracts from 400 DAS at 4°C. Moreover, this stabilization effect was not observed with tall fescue extracts from short term cold stress or control conditions samples (Fig 8, upper panel). To further investigate the enzyme stabilization in barley caused by tall fescue extract, we assessed different treatments on barley extract mixing with crude, dialyzed or boiled tall fescue extract obtained from samples at 400 DAS under cold stress (Fig 8, lower panel). Amylolytic activity did not show differences in samples were barley extract was mixed with crude and boiled tall fescue extract, whereas the amylolytic activity obtained by mixing dialyzed tall fescue extract with barley extract presented analogous pattern obtained by the barley extract alone (Fig 8, lower panels).

## Discussion

In the present study, a comprehensive insight into the physiological response of tall fescue to prolonged starvation was obtained. The species proved to have an extraordinary capacity to survive extremely long periods under nutritional and cold stresses. Temperature variation is one of the most pervasive environmental influences on higher plants, with instantaneous effects on the tissue dynamics in the elongation zones [34]. Previous studies reported that temperature affects tall fescue caryopses germination in terms of vigor, speed and rate [16, 18–20]. Interestingly, our analysis of coleoptile and root elongation revealed a 10-fold delay in germination and growth in cold treated plants, indicating that growth is primarily restricted by thermodynamic limitations as occurred in other species such as wheat and rape [35].

Our study showed that the ability of tall fescue to sense low temperature stress determines the activation of cold acclimation mechanisms during germination and development that lead to modified cells structure in the seedling. These modifications include greater thickening of the cell wall, presence of undifferentiated plastids, increase of root hairs and reduce lignification of xylem cells. Analogous anatomical adaptations have been observed in rice [36]. Moreover, non-coordinated progression between cell division or cell growth and the development of undifferentiated plastids to etioplasts seems to play a key role in the induction of cool-temperature-induced chlorosis (CTIC) in rice, which can also determine significant growth retardation (Yoshida et al. 1996). Accordingly, tall fescue caryopses grown under cold temperature showed severe growth retardation and seedlings remained alive as long as to 400 DAS without succumbing, suggesting the induction of CTIC-like phenomenon. In addition, we found that transfer 400 d old tall fescue plants from 4°C/dark to 23°C/light triggered the progression of etioplasts to normal chloroplasts, suggesting that in tall fescue the CTIC-like phenomenon may be reversible with a subsequent normal expression of plastid-encoded genes.

Our data highlight the opposing role of ABA and GA in seedling growth and development in accordance with previous reports (Karssen and Laçka 1986; Bewley 1997). Previous study revealed that although ABA plays a critical role in plant stress response, cold stress does not induce *per se* a significant level of ABA in plants [37]. However, ABA does appear to be necessary to maximize the induction of cold-responsive genes, and thus, cross-talk between cold and ABA signaling pathways may exist [38]. Moreover, Dierking and Kallenbach [9] observed that the accumulation of ABA in response to cold stress in tall fescue occurs to a lesser degree than what is observed in response to other abiotic stresses. All evidences support our results under long-term cold stress, where ABA showed predominance in embryos according to the delayed germination, while coleoptile and roots did not showed significant increase of ABA in response to cold stress. GA is another phytohormones involved in abiotic stress responses in plants [39]. Previous study in pea revealed that GA could act as a mediator of the thermoperiodic response, reducing its stem elongation with the decrease of GA_1_ [40]. Consistent with this, our data suggests that altered coleoptile and root elongation of tall fescue plants in response to temperature alternations may be mediated by changes in the endogenous GA level, which was lower in plants grown at long-term cold stress than in that of control conditions.

In cold hardy herbaceous species, long-term exposure to low temperature results not only in the accumulation of large pools of soluble carbohydrates [41], but also in the enhancement of pathways involved in photosynthetic carbon metabolism [35]. In this light, the carbohydrate accumulation observed at 120 DAS in all tissues of cold treated seedlings was to be expected, perhaps with a similar amplitude to that previously observed by Dierking and Kallenbach [9] on Mediterranean and Continental tall fescue accessions exposed to 4.5°C for 30 d. Carbohydrates content started to decline from 120 DAS up to 400 DAS plants at 4°C, similar to 21 DAS plants at 23°C, indicating that this gradual sugar starvation happened due to its use in basal metabolism during long-term cold stress. Moreover, the modulation of carbohydrates balance in germination response to long-term cold stress could regulate genes involved in starch degradation observed in our results, as previously reported in cereal embryos [8].

Tall fescue maintained its vitality under full starvation stress as long as 400 DAS, which testifies to its remarkable ability to cope with prolonged stress. In this study, we observed the presence of distinct stable α-amylase isoforms at long-term cold and nutritional stresses, which correlate with the complete starch depletion. This coordinated change in levels of α-amylase activity under long-term starvation conditions suggests that these proteins may be physiologically relevant, with a convergence of nutritional and environmental stress signal transduction pathways that trigger a specific sequence of events in plant cells [3]. Moreover, we could verify that long-term starved tall fescue stabilizing factors could also stabilize the amylolytic activity in barley. This evidence prompts us to speculate that small molecules, possibly Ca^2+^, may be responsible for the stabilization of the amylase isoforms. Ca^2+^ plays crucial role in converting external signals into cytosolic information, driving processes that are mandatory in response to environmental stimuli, such as low temperatures (Catalá et al. 2003; Kim et al. 2003). Biochemical studies have highlighted how higher plants encode a particularly large superfamily of calcium-dependent PKs (CDPKs) which are involved in intricate pathways (Rolland et al. 2006). In addition, Ca^2+^ also affects the process of enzyme synthesis and transport, and is required to maintain both the activity and the stability of the α-amylases secreted by the aleurone layer [8, 42]. Altogether, Ca^2+^-mediated regulation pathways in tall fescue are likely required in response to long-term starvation stress. Further studies are needed to understand the detailed mechanism of tall fescue in response to long-term starvation stress.

## Conclusions

The present work functionally characterizes tall fescue germination and seedling development in response to combined nutrient and cold stresses. In this work, tall fescue is further validated as a model species for physiological studies of thermal adaptation as it expresses strong persistency and high tolerance to starvation stress. Convergence of starvation signals and hormone signals meet in crosstalk to regulate α-amylase activity upon germination and seedling development in tall fescue, with stable amylase isoforms being induced by prolonged famine.

## Supporting Information

S1 Table Results of shoot and root elongation, endogenous level of ABA and GA in caryopses, in coleoptile and root tissues, starch and total soluble sugars content in endosperm, coleoptile, and root tissues, and amylolytic activities in tall fescue (*Festuca arundinacea* Schreb.) caryopses grown under dark at 23°C and 4°C. (XLSX)
